# Extractable and Non-Extractable Antioxidants Composition in the eBASIS Database: A Key Tool for Dietary Assessment in Human Health and Disease Research

**DOI:** 10.3390/nu12113405

**Published:** 2020-11-06

**Authors:** Jenny Plumb, Alessandra Durazzo, Massimo Lucarini, Emanuela Camilli, Aida Turrini, Luisa Marletta, Paul Finglas

**Affiliations:** 1 Quadram Institute Bioscience, Norwich, Norfolk NR4 7UQ, UK; paul.finglas@quadram.ac.uk; 2 CREA—Research Centre for Food and Nutrition, 00178 Rome, Italy; massimo.lucarini@crea.gov.it (M.L.); emanuela.camilli@crea.gov.it (E.C.); aida.turrini@crea.gov.it (A.T.); luisa.marletta@crea.gov.it (L.M.)

**Keywords:** eBASIS, extractable compounds, non-extractable compounds, bioactive compounds, human health, dietary intake, dietary assessment, antioxidant properties, nutrition knowledge

## Abstract

The antioxidant properties of foods are crucial in nutrition, food chemistry, and medicine studies but are often underestimated, with significant amounts of bioactive compounds containing physiological and biochemical properties remaining in the residue from extraction as non-extractable antioxidants. Over the last decade, extractable and non-extractable compounds have become key in the evaluation/determination of the antioxidant properties of food matrices because of their relevance in human health. This has led to the need to include extractable and non-extractable antioxidants in comprehensive and harmonized food composition databases for a wide range of applications within research, food, pharmaceutical, nutraceutical, and cosmeceutical areas. Additionally, the databases are invaluable as part of the health claims application process. eBASIS, (Bioactive Substances in Food Information System) a comprehensive database containing quality-evaluated scientific data, covering the composition of bioactive compounds present in foods, has flexible structures, allowing it to be extended to include newly emerging data on extractable and non-extractable compounds. Search criteria were developed and defined for compiling suitable peer-reviewed literature. Data quality assessment methods were established for the addition of composition data and antioxidant activity, with a focus on various parameters including: the extraction procedure, the antioxidant measurements, the expression of results. A total of 437 quality-evaluated datapoints on the composition of extractable and/or non-extractable compounds were entered into the database. This database update represents one of the first examples of building a database dedicated to antioxidant properties. This expansion of eBASIS provides a novel and unique tool for nutritionists, dietitians, researchers to use for a wide range of applications, such as dietary assessment, exposure studies and epidemiological studies, and may contribute to an increase in high-bioactive food consumption by consumers.

## 1. Introduction

Over the last decade, growth in the interest of bioactive compound research [1,2,3,4] has expanded to include extractable and non-extractable compounds, which are key in the evaluation/determination of the antioxidant properties of food matrices [5,6,7,8,9]. A recent study by Yeung et al. [9], carrying out a scientific literature landscape analysis, showed that scientific interest has recently expanded to include research on antioxidant phytochemicals. This has led to the emergence of extractable and non-extractable polyphenols as a key tool in the description of the antioxidant properties of food [10,11]. It follows that there is a requirement for the inclusion of extractable and non-extractable antioxidants into comprehensive and harmonized food composition databases for a wide range of applications, such as dietary intake assessment [10,11].

### 1.1. The Main Feautures of Extractable and Non-Extractable Antioxidants

#### 1.1.1. Chemistry

Extractable and non-extractable antioxidants are classified by their methods of extraction as follows: easily extractable polyphenols (free forms) (EPP) are solubilized by aqueous-organic solvents, whereas less extractable polyphenols are bound forms, remaining in the residue of aqueous-organic extract [12].

Extractable polyphenols comprise low-molecular-weight compounds from several classes and subclasses of polyphenols, including proanthocyanidins (dimers and trimers) (Extractable proanthocyanidins—EPA) and hydrolysable tannins of low molecular weight.

In contrast, non-extractable polyphenols (NEPP) remain in the residues of the previous extractions and can only be released by hydrolysis treatments, either chemical or enzymatic. NEPP consist of:

(a) Polymeric polyphenols (non-extractable proanthocyanidins NEPA), high-molecular-weight proanthocyanidins that are free in the food matrix and proanthocyanidins that are complexed with protein or cell wall polysaccharides [13];
(b) Small phenolic compounds, linked to carbohydrates, mainly polysaccharide constituents of dietary fibre, and to proteins (hydrolysable polyphenols; HPP). These include several classes of bioactive components, e.g., hydrolysable tannins or hydroxycinnamic acids, linked to carbohydrates and proteins via covalent bonds, hydrogen bonds and/or hydrophobic interactions [14,15].

While extractable polyphenols are obtained using aqueous-organic solvents, different hydrolytic treatments of the residue specific are performed for the isolation of specific fractions of non-extractable compounds, i.e., HPP and NEPA. Extraction and analysis procedures are developed and performed in relation to the food item within each food group, the type of bonds with the food matrix as well as the nature and structure of the target compounds [15,16,17]. Recently, the scientific community has reached consensus on the distinction between extractable and non-extractable antioxidants, and development and assessment of methodologies was achieved [10].

#### 1.1.2. Health Benefits and Role in the Prevention of Chronic Diseases

The complex relationship between food, nutrition, and health requires detailed knowledge of the composition of nutrients and bioactive compounds. Studies on antioxidant properties is one step in defining the potential beneficial properties of foods, as well as for studies understanding physiological mechanism and bio-accessibility. In this regard, in vitro and in vivo studies on the exploitation of health effects of extractable and non-extractable antioxidants are being carried out [18,19,20,21,22]. For example, the work of Cheng et al. [18] studied the effects of extractable and non- extractable compounds isolated from blueberries using an in vitro model of inflammation: both fractions exerted anti-inflammatory properties throughout the inhibition of iNOS and COX-2 mRNA expression through the suppression of NF-kB. Additionally, Gonzalez-Sarrías et al. [22] documented how NEPP produce gut microbiota metabolites that persist in circulation and present anti-inflammatory and free radical scavenging activities. The work of Han et al. [20] demonstrated how EPP and NEPP fractions from cranberries were bioactive, and particularly the NEPP fraction, presented promising anti-inflammation and anti-colon-cancer potential: NEPP showed stronger inhibitory effects on the viability and colony formation capacity of human colon cancer HCT116 cells than the EPP; the authors [20] also described, how in a flow cytometry analysis, NEPP caused cell cycle arrest at the G0/G1 phase and induced significant cellular apoptosis in colon cancer cells. Another recent work of Hamauzu and Suwannachot [21] reported stronger bile acid-binding activity of the NEPP from dried persimmon (*Diospyros kaki*) fruit.

This research indicates the potential utilization of extractable and non-extractable antioxidants using an integrated approach, based on a combination of studies from different fields (nutrition, food chemistry, medicine, etc.).

#### 1.1.3. Occurrence in Foods and Dietary Intake Assessment

The antioxidant properties of foods are often underestimated, with significant amounts of bioactive compounds containing physiological and biochemical properties remaining in the residue from extraction as non-extractable antioxidants [14,15,16,17,23,24]. The incidence and the distribution/ratio of EPP, EPA, NEPP, NEPA, HPP have been studied in raw, cooked, and processed foods [8,12,25,26,27,28,29,30,31,32,33,34,35,36,37,38,39,40,41,42]. Due to the presence of multiple aspects and factors (variability of type of food matrix within each food group, part of food matrix, cultivar/species, growing factors, technological process, formulation, etc.), it is difficult to carry out a categorization of the main trends of the contribution of extractable and non-extractable compounds to the total antioxidant properties of the major food groups. Generally, however, researchers conclude that the analysis of antioxidants in foods that remain in the residues is necessary for a comprehensive and appropriate identification of antioxidant properties [23]. Currently, the development of methodologies, with particular regard to green ones, in the use of antioxidant properties from food wastes as part of a bio-economy system and circular economy, is being researched [43,44,45,46,47].

Moreover, the assessment of the contribution of extractable and non-extractable compounds to dietary intake is a key issue that is being carried out in several works [48,49,50,51,52,53].

Saura-Calixto et al. [48] estimated the amount of total polyphenols (in terms of extractable and non-extractable polyphenols) consumed in a whole diet (Spanish Mediterranean diet) and also evaluated their intestinal bioaccessibility: the amount of non-extractable polyphenols was double that of extractable polyphenols. Pérez-Jiménez and Saura-Calixto [51] studied non-extractable polyphenols of the 24 most consumed fruit and vegetables in four European countries (France, Germany, The Netherlands, and Spain): the major contributors (mean value 57%) to the total polyphenol content of fruit and vegetables are macromolecular antioxidants, consisting of hydrolysable polyphenols and polymeric proanthocyanidins, and the estimation of daily per capita intake of non-extractable polyphenols, was about 200 mg.

Recent work by Koehnlein et al. [50] estimated the total dietary antioxidant capacity (TDAC) in the Brazilian population: TDAC, determined as the ferric-reducing antioxidant power and as the Trolox equivalent antioxidant capacity, was reported to be 10.3 and 9.4 mmol/d, respectively. Further work [52] from the same authors documented the comparison of phenolic content and the total antioxidant capacity of the 36 most popular Brazilian foods submitted to aqueous extraction with in vitro digestion, showing higher phenolic contents and higher antioxidant activities for cereals, legumes, vegetables, tuberous vegetables, chocolate, and fruits after in vitro digestion, compared to those obtained by aqueous extraction. The digestion caused a reduction in phenolic contents and the antioxidant activities of beverages (red wine, coffee, and yerba mate) [52].

Interest in the human gut microbiome has become a major research area in recent years, and a study by Faller et al. [49], studying the chemical and cellular antioxidant activity of feijoada, a typical Brazilian dish, coupled with an in vitro digestion model, has suggested that phenolics are capable of reaching the colon after the intake.

### 1.2. Development of Dedicated Databases on Antioxidant Properties

This recent interest in extractable and non-extractable compounds, which are key in the evaluation/determination of antioxidant properties of food matrices, and the lack of dedicated databases on antioxidant properties has led to the need for data to be compiled and stored in a quality evaluated, harmonized database. The overall goal, representing the novelty character research, is to have access to such a dedicated food composition database in order to be able to estimate an adequate dietary intake evaluation.

In developing a database dedicated to antioxidant properties, it is essential that certain parameters are taken into consideration to create a reliable, quality evaluated resource. These include extraction procedure, antioxidant measurements and expression of results [54,55,56,57,58]. The issues around these parameters are highlighted below:

- Extraction procedure—the method of extraction is key, including different procedures and different solvents and/or mixtures thereof [54,59,60], with recent studies performing alkaline hydrolysis, acid hydrolysis or enzymatic digestion [61,62,63,64,65];
- Antioxidant measurements—several different assays are performed to evaluate the antioxidant properties of single compounds and foods, these differ in their principles, mechanisms, and experimental conditions, as well as in the way end points are measured [66,67]. For high-quality data, the use of at least two or three assays is strongly recommended [66,68];
- Expression of results—there are different ways of expressing antioxidant results, including kinetic parameters [16].

Historically, some approaches to creating specific databases on antioxidant properties have been carried out [69,70], however, these studies are limited by considering only a single assay, not using common extraction procedure or neglecting the extraction method used.

Therefore, the inclusion of quality-evaluated data on extractable and non-extractable antioxidants, as part of the eBASIS structure, creates, for the first time, a valid new tool for the food, nutraceutical, pharmaceutical and cosmeceutical fields, by reflecting a reliable, novel and more representative approach for assessment antioxidant properties, and indeed healthy properties, in databases.

### 1.3. eBASIS

The Bioactive Substances in Food Information System (eBASIS) is a web-based database that contains validated scientific information on the composition of bioactive substances in plant foods [71]. The database has been developing since its launch in 2006 [72], with a user-friendly, efficient, and flexible interface facilitating used by both the scientific community and food industry. The need for easily accessible information on the composition, intake, and activity of extractable and non-extractable antioxidants is crucial for researchers, hence, the need for data provided in a convenient and widely accessible form, producing a valuable unique resource.

The database combines bioactive composition data of 24 compound classes, e.g., glucosinolates, phytosterols, polyphenols and isoflavones in around 300 major European plant foods [73,74]. The eBASIS resource represents a collection of data from the peer-reviewed literature, evaluated critically by experts and inserted as raw data; the flexible structure is designed to allow the addition of new compounds and foods, for example the inclusion of bioactive peptides in meats [74], and, for this update, extractable and non-extractable compounds. This paper provides an overview on the first development of a database dedicated to antioxidant properties, included as part of the eBASIS resource.

## 2. Materials and Methods

### 2.1. eBASIS

eBASIS is hosted by EuroFIR AISBL, with access via membership packages or within EU projects (http://ebasis.eurofir.org). It is a relational database served by Microsoft Windows Server version 2008 R2 Enterprise, Microsoft Internet Information Services version 6.1, and Microsoft SQL Server version 2008 (Microsoft, Redmond, WA, USA), which is also verified to operate on version 2012. eBASIS composition data are managed by Quadram Institute Bioscience, UK, with entry carried out by trained evaluators and all inputs checked by database managers before appearing in the database. The data entry goal is to source, extract, and quality-assess data from peer-reviewed publications satisfying pre-defined quality criteria. The data input form is designed to promote consistency in data entry, with 35 fields used to collect composition data. Table 1 shows the categories and types of data collected. The forms are designed to be unambiguous and simple to use and come with clear instructions. Where possible, pick lists are used, which also enable detailed searches to be carried out by database users and free text fields intentionally limited to simplify reporting and data analysis of eBASIS contents. Publications are sourced and assessed through standardized search and selection protocols, and each food–compound combination is entered separately. Standard operating procedures (SOPs) are followed in each step of the compilation process [72].

### 2.2. eBASIS Revisions to Expand the Database for Antioxidants

Originally, eBASIS was designed for plant and individual bioactive component composition data, therefore, some structural changes were required to adapt the database to this new information whilst maintaining an appropriate structure and functionality.

eBASIS uses online forms to enter quality-evaluated data using a systematic approach, described by Gry et al. [72] and documented in Table 1. The new data required for this update required inclusion of the following classes:

Extractable polyphenols (EPP);
Extractable proanthocyanidins (EPA);
Non-extractable polyphenols (NEPP);
Non-extractable proanthocyanidins (NEPA);
Hydrolysable polyphenols (HPP).

eBASIS, to this date, deals with composition units as mg/kg or µmol/kg, fresh or dried weight. Using standard units allows the user to compare and aggregate data, however, this update required new units for the data to be included to allow the addition of data sourced from peer-reviewed publications, similarly, the expansion of analytical method options was required. Examples of types of analytical methods and units included for composition and activity information are:

- The content of antioxidant EPP/EPA/NEPP/NEPA/HPP: the data are entered as documented in the original publication, e.g., milligrams of EPP/NEPP/EPA/NEPA/HPP per kilogram of food in fresh or dry weight;
- Antioxidant activity properties evaluated by 2,2 -azino-bis(3-ethylbenzothiazoline-6-sulfonic acid) (ABTS), 2,2-Diphenyl-1-picrylhydrazyl radical (DPPH), Ferric Reducing Antioxidant Power (FRAP), Oxygen Radical Absorbance Capacity (ORAC) and Folin-Ciocalteu assays: since units will vary for the contribution of EPP/EPA/NEPP/NEPA/HPP, it was essential to add new units to eBASIS. Examples include: ABTS assay data reported as mM trolox per kilogram of food, FRAP assay expressed as mM Fe_2_SO_4_ per kilogram of food, and ORAC assay data expressed as mM trolox per kilogram of food.

### 2.3. Search Criteria and Suitable Papers for Data Entry

Systematic literature searching for composition data remained largely the same as carried out during eBASIS, as described by Gry et al. [72], and Plumb et al. [75]. Search criteria were defined, and bibliographic research carried out on search engines: Pubmed, Scopus, Science Direct. The following keywords as search criteria were adopted: extractable polyphenols, non-extractable polyphenols, hydrolyzable polyphenols, hydrolyzable tannins, extractable proanthocyanidins, non-extractable proanthocyanidins.

Papers, including procedures to obtain extractable and/or non-extractable compounds, were evaluated for data entry. Sourced papers with data express in graphical format; data on non-edible plants only; data on multi ingredient processed foods were rejected for data entry.

Since the use of an adequate assay for antioxidant properties depends on the class compounds/fractions and the type of food matrix, the suitability of assays for data entry was evaluated for each paper. Particular attention was given to the profile/distribution of compounds within each fraction.

**Figure 1:**
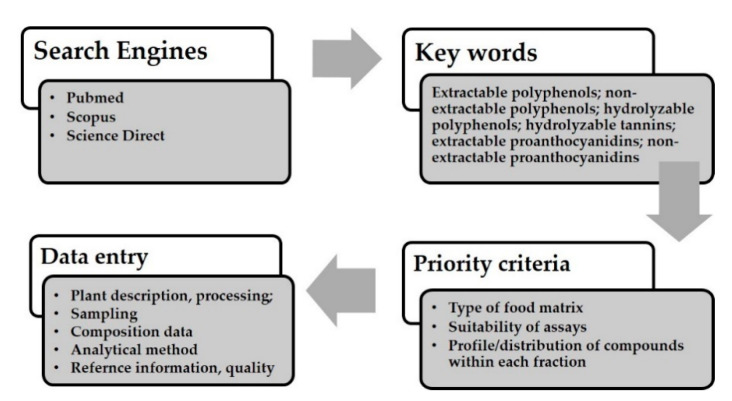
Flow diagram showing selection of studies for data entry.

Common elements of papers to guarantee suitability for data entry were clarified, following this, data entry methods for addition of composition data and antioxidant activity of extractable and non-extractable compounds were defined. The flow diagram for the selection of studies is reported in Figure 1.

Subsequently, data entry was tested on selected papers, and proposals for improved database structure and function for data inputting and reporting were produced. Data quality assessment methods were established for the addition of composition data and antioxidant activity with a focus on various parameters including: the extraction procedure, the antioxidant measurements, the expression of results [54,58].

## 3. Results and Discussion

### 3.1. Test Input Systems for Data Entry

Two input form models were formulated: one using the current eBASIS fields, another to develop modifications in eBASIS structure, such as the revision of field names, to allow for a clearer separation of composition and activity information.

**Figure 2:**
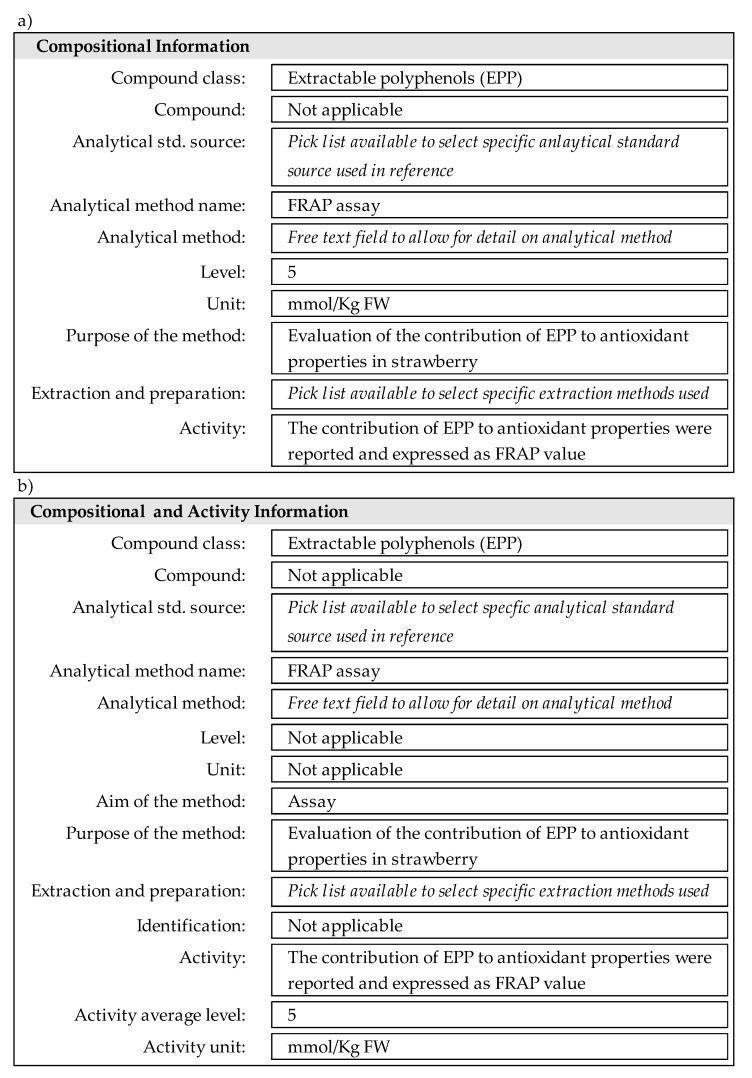
(**a**) Composition information section of eBASIS input form for data entry for EPP; (**b**) proposed model for input form for EPP, in fresh weight (FW) material, using Ferric Reducing Antioxidant Power (FRAP) assay.

For data entry using current eBASIS fields, food information (including plant, part, species, country of origin), processing (cooking method, treatment, preservation method), sampling information (year, primary sample size, analytical sample size, sample plan, sampling handling) and quality assessment (food description, processing defined, sampling plan, sample handling, analytical method and analytical performance) remain the same (see Figure 2). For the compositional information section in the input form, different classes of extractable and non-extractable antioxidants were added to the compound class pick list: extractable polyphenols (EPP), extractable proanthocyanidins (EPA), non-extractable polyphenols (NEPP), non-extractable proanthocyanidins (NEPA), hydrolysable polyphenols (HPP). In addition, new analytical methods were included within the pick list, i.e., FRAP assay, DPPH assay, ORAC assay, ABTS assay, Folin-Ciocalteu assay.

As examples of part of the input form for eBASIS, Figure 2a shows how compositional data are entered for EPP. This demonstrates compositional input showing the contribution of extractable polyphenols (EPP) to antioxidant properties in strawberry, evaluated by means of FRAP assay, resulting in a FRAP value of 5 mmol/Kg FW for EPP.

For the new data, the eBASIS quality assessment remained the same, with peer-reviewed data critically evaluated across a number of key areas: food description; processing; sampling plan; sample handling; compound identification; analytical method; and analytical performance.

The input forms were tested and used for data entry of EPP and NEPP in eBASIS. Moreover, the new help text to clarify the criteria for data entry methods, as well as a short guide for databases users were created.

Future modifications in the eBASIS structure for a clearer separation of composition and activity information were deemed necessary, with a proposal to improve database structure and function developed to help data entry and data reporting. Models for the improved input forms, including new fields, referred to the above EPP example, shown in Figure 2b. This includes proposed additional fields separating activity levels from the current individual compound levels; “compositional Information” replaced by “compositional and activity information” and additional fields “activity average level”, “activity minimum level” “activity maximum level”, “activity standard deviation”, “activity unit” should be included.

Further adjustments to the database will be required when reporting levels of HPP and EPP analysed by DPPH assay. Four papers retrieved for data entry reported composition values in the form of g/g DPPH, leading to 15 values that could not be included using current eBASIS inputting systems.

### 3.2. Producing eBASIS Reports

**Figure 3:**
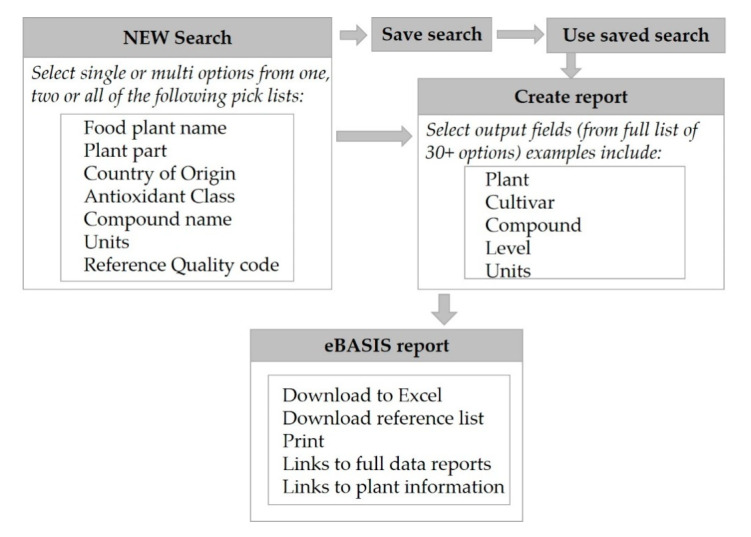
Creation of eBASIS reports.

Methods for searching for, and extracting, the new composition and/or activity data contained in eBASIS was improved and enhanced during this update. Database searching is user-led through a variety of parameters: antioxidant class, food, or a combination of both class and food. The level of detail viewed is also user-led; default fields are provided with the option for the user to include additional fields, including reference, analytical method name, or sampling information. The report, once produced, can be downloaded and manipulated in spreadsheets, and the list of references used to compile the individual report can be viewed and saved for inputting into reference library software. Search criteria can also be saved for further use. Figure 3 shows the eBASIS systems for searching and reporting.

Creating reports and searching for the newly added data is easy and simple. Composition and activity data are referred to and searchable via compound class: extractable polyphenols (EPP), non-extractable polyphenols (NEPP), extractable Proanthocyanidins (EPA), non-extractable proanthocyanidins (NEPA), hydrolysable polyphenols (HPP). The compound field, used traditionally for bioactive compound databases, is not applicable to the new data, however, including the analytical method in search options is essential for comparison of data, i.e., FRAP assay, DPPH assay, ORAC assay, ABTS assay, Folin-Ciocalteu assay.

### 3.3. Data on Extractable and/or Non-Extractable Compounds, from Peer-Reviewed Literature

As part of this update of eBASIS, 437 new, quality-evaluated datapoints have been added, from 27 peer-reviewed literature sources’ data; this includes data on EPP, EPA, NEPP, HPP and NEPA in 63 different edible plants (Table 2 and Supplementary Materials Table S1) report. The plant foods with compositional data include a range of fruits, vegetables and pulses as well as processed foods such as chocolate and brewed green tea. The 27 references containing data added in eBASIS range from single publications each year in the period 2005–2011, to 15 within the last 4 years, showing an increase in scientific interest in this area. These new data become a subset of the full eBASIS database which contains quality-evaluated data from 1300 peer-reviewed publications, yielding 44K datapoints on the composition of 794 individual bioactive compounds in 270 food plants. Each datapoint is a unique entry for a specific plant/compound combination, and a single publication may yield from one, to over 100 records.

## 4. Discussion

### 4.1. Data on Extractable and/or Non-Extractable Compounds Exploitation

This new updated compilation of quality-evaluated data of food plant extractable and non-extractable compounds, alongside current contents of eBASIS, represents the need for such a useful tool for a wide range of applications. Examples of such applications include assessing and improving databases for nutrition and health applications, dietary intake assessment, exposure studies, epidemiological studies and clinical trials [76,77,78]. Industries, including food, pharmaceutical, nutraceutical and cosmeceutical, may find it invaluable as part of the health claims application process [73,79,80].

The database holds a massive resource of data that may be used to quantify definable biochemicals present in our food [81]; currently, it is being used within the European Union’s Horizon 2020 Research and Innovation programme, ‘Food Nutrition Security Cloud’ (FNS-Cloud) [82], to aggregate data on bioactives in a range of plant-based foods in order to create high- and low-bioactive content diets for human intervention studies, with the goal of better understanding the relationship between diet and health.

### 4.2. Bioactive Composition Data Aggregation for Dietary Assessment

The most common application of food composition data in research is in combination with dietary intake data to provide estimates of nutritional exposure or intake, required for both large-scale epidemiology studies and for smaller interventions [83]. These dietary assessment studies require databases or tools with a detailed coverage of foods and nutrients, however, there is currently a lack of integration with dietary data on bioactive composition, including the extractable and non-extractable compounds. In order to integrate databases for nutrients and non-nutrients, there needs to be continued efforts to standardise the bioactive data, with a goal to produce aggregated, standardised findable, accessible, interoperable and reusable (FAIR) bioactive food composition data.

eBASIS currently provides users with the ability to export to excel in order to manipulate the data for their own use. eBASIS does not aggregate data; it was assumed, when the database was developed, that users would wish to see all data, including ranges of bioactive composition between different varieties of the same plant, plant part, growing conditions, processing and country of origin. This, however, is its secondary use compared to the common requirement for aggregated bioactive composition data for use in intake studies. This has led to the requirement to aggregate the data to produce a single figure for a plant/compound combination while considering plant part, recorded units, and any food processing. The ideal bioactive aggregation would create a figure for the mean, median, minimum and maximum composition, and have the ability to find the source of all relevant references, to see, at a glance, the amount of inputs used and list all the inputs that were used to create the aggregation.

To overcome the current challenge within eBASIS where reports produce multiple datapoints for each compound/food combination, detailed work is underway to create aggregated data that can be added to standardised food composition databases, ensuring food codes are comparable and data can be easily retrieved in support of pan-EU food and health studies. The final objective is to include bioactive data within apps, dietary assessment systems and international food composition tools such as FoodCASE (a food composition data management tool) and FoodEXplorer (a query tool of food composition data across 37 countries) [84].

This aggregation update is in progress in eBASIS with a launch, planned in 2021, of a new bioactive composition database which can feed directly into dietary assessment within multi-disciplinary national and international research projects in the areas of epidemiology, diet and health, and emerging research infrastructure initiatives. These modifications of eBASIS structure additionally allow for a clearer separation of composition and activity information, leading to the potential to have a standalone database dedicated to Antioxidant Properties.

## 5. Conclusions

A relevant number of datapoints on the composition of extractable and/or non-extractable compounds have been entered into the eBASIS database. This new database update is intended to be a first example of building a database including extractable and non-extractable compounds and expression of antioxidant properties. Moreover, the description of input procedures could be a useful tool/guide for other compilers and users. eBASIS continues to be one of the major databases on composition of bioactive compounds in foods, with all of the data traceable to original peer-reviewed publications. This update creates a comprehensive instrument that includes compiled information on antioxidant compounds and antioxidant properties. Alongside this, the inclusion of transparent quality systems makes it an important, reliable resource for research with ongoing sourcing and data entry on extractable/non extractable phytochemicals.

## Figures and Tables

**Table 1:** eBASIS data entry fields.

**Reference**	**Food**	**Processing**
Author Title Journal Year, Volume Summary of paper contents	Plant, part, cultivar, maturity, country of origin, region, season, growing conditions, EuroFIR classification, generic food name, plant description	*If food is processed, these fields are included* state, heat treatment, cooking method, treatment applied, preservation method
**Sampling**	**Composition**	**Quality**
Sampling year, primary sample unit size, primary sample units, analytical sample size, portion replicates, sample plan, sample handling	Compound class/activity, compound, standard source, analytical method name, analytical method, level, min, max, standard deviation, unit, extraction method	Plant description, processing defined, sample plan, sample handling, compound identification, analytical method, analytical performance

**Table 2:** Data included within eBASIS database.

	Data Points	Number of Food Plants	References
EPP	187	44	23
EPA	11	3	2
NEPP	36	5	5
NEPA	88	27	10
HPP	115	48	15
Combined total	437	63	27
**eBASIS total**	**44,300**	**271**	**1300**

## Supplementary Materials

The following are available online at https://www.mdpi.com/2072-6643/12/11/3405/s1. Table S1. eBASIS report 2020.
